# Characterisation of recombinant CD23 in the trimeric complex with IgE and allergen

**DOI:** 10.1186/1939-4551-8-S1-A192

**Published:** 2015-04-08

**Authors:** Regina Maria Selb, Julia Eckl-Dorna, Christian Lupinek, Birgit Linhart, Andrea Teufelberger, Walter Keller, Kenneth H Roux, Rudolf Valenta, Verena Niederberger

**Affiliations:** 1 Medical University of Vienna, Austria; 2 Karl Franzens University, Austria; 3 Florida State University, USA

## Background

CD23, the low affinity receptor for IgE, is mainly expressed on B cells and plays an important role in allergy. Besides its possible role in the regulation of IgE production, CD23 plays an important role in IgE-facilitated allergen presentation to T cells and subsequent activation of allergen-specific T cells.

## Methods

Four CD23 protein versions were recombinantly expressed in SF9 insect cells, human monoclonal IgE was isolated from a hybridoma cell line.

## Results

The characterisation of CD23 via circular dichroism (CD) spectra and gel filtration showed folded, monomeric proteins. Binding of isolated monomeric human monoclonal IgE and of isolated polyclonal serum IgE as well as of both forms of IgE in complex with birch pollen allergen Bet v 1 to recombinant CD23 was demonstrated by ELISA and by surface plasmon resonance analysis.

Next, we performed negative stain electron microscopy of the three molecules alone (i.e., CD23, monoclonal human IgE, Bet v 1) and after complex formation. After addition of Bet v 1 allergen (17 kDa) to monoclonal IgE (190 kDa) we could observe an extension of one or both Fab arms of the antibody. Interestingly, further addition of recombinant CD23 molecules (35 kDa) to the IgE-allergen complex resulted in thickening of the antibody’s Fc structure, possibly because CD23 lies in the same plane as the IgE molecule in these pictures.

## Conclusions

In summary, we report the *in vitro* formation of a trimolecular complex consisting of recombinant CD23, a monoclonal human allergen-specific IgE and the corresponding Bet v 1 allergen and take a first step towards the visualization of this complex using negative stain electron microscopy. Furthermore, the *in vitro* trimolecular interaction model may be useful for the screening of drugs and compounds for their potential to inhibit the IgE CD23 interaction with the goal to develop new therapeutic strategies for allergy.

## Acknowledgements

Supported by grants 4605, 4613 and in part by 4604 and P23350-B11 of the Austrian Science Fund (FWF).

